# ﻿A new species of the genus *Ovophis* Burger in Hoge & Romano-Hoge, 1981 (Serpentes, Viperidae) from southern Yunnan, China

**DOI:** 10.3897/zookeys.1230.142967

**Published:** 2025-03-06

**Authors:** Shuo Liu, Mian Hou, Mingzhong Mo, Mei Li, Biao Li, Xiong Luo, Dingqi Rao, Song Li

**Affiliations:** 1 Kunming Natural History Museum of Zoology, Kunming Institute of Zoology, Chinese Academy of Sciences, Kunming, Yunnan 650223, China; 2 College of Continuing (Online) Education, Sichuan Normal University, Chengdu, Sichuan 610066, China; 3 Honghe Prefecture Forestry and Grassland Bureau of Yunnan Province, Mengzi, Yunnan 661199, China; 4 Yuanyang Guanyinshan Provincial Nature Reserve Management and Protection Bureau, Yuanyang, Yunnan 662400, China; 5 Kunming Institute of Zoology, Chinese Academy of Sciences, Kunming, Yunnan 650201, China; 6 Yunnan Key Laboratory of Biodiversity Information, Kunming Institute of Zoology, Chinese Academy of Sciences, Kunming, Yunnan 650201, China

**Keywords:** cytochrome *b* gene, morphology, mountain pitvipers, systematics, taxonomy

## Abstract

A new species of *Ovophis* is described from Yuanyang Guanyinshan Provincial Nature Reserve in southern Yunnan Province, China. The new species can be distinguished from congeneric species by the following combination of characters: ratio of tail length to total length 0.191–0.206, internasals separated by one or two scales, dorsal scales in 22-21-17 rows, ventrals 146–148, subcaudals 57–64, most subcaudals paired and a few unpaired, third supralabial larger than fourth, white spots on dorsal tail continuous. This study further reveals that the species diversity of *Ovophis* has been seriously underestimated, and there is still a lot of work to be done on the taxonomy of this genus.

## ﻿Introduction

The genus *Ovophis* Burger in Hoge & Romano-Hoge, 1981 is a group of medium-sized venomous snakes that are widely distributed in mountainous areas of eastern Asia, southern Himalayas, and Indochina (Leviton et al. 2003; Neang et al. 2011; Che et al. 2020; Guo et al. 2022; Guo and Che 2024). Currently, this genus is recognized to contain seven species, namely *O.monticola* (Günther, 1864), *O.convictus* (Stoliczka, 1870), *O.makazayazaya* (Takahashi, 1922), *O.tonkinensis* (Bourret, 1934), *O.zayuensis* (Jiang, 1977), *O.anitae* David, Frétey & Vogel, 2024, and *O.jenkinsi* Qiu, Wang, Xia, Jiang, Zeng, Wang, Li & Shi, 2024 (Qiu et al. 2024). In addition, *Trimeresurusokinavensis* Boulenger, 1892 and *Trimeresurusgracilis* Oshima, 1920 may also belong to this genus since they share similar morphological characteristics with the above seven species (Uetz et al. 2025). However, these two species were not supported in *Ovophis* based on molecular data (Malhotra and Thorpe 2000, 2004; Li et al. 2020; Shi et al. 2021). Therefore, recent taxonomic studies did not include these two species in *Ovophis* (e.g., Zeng et al. 2023; Qiu et al. 2024).

Previously, *Ovophismonticola* was considered a widely distributed species, and the snakes of this genus occurring in Yunnan Province of China were all considered to be *O.monticola* (Zhao et al. 1998; Zhao 2006; Yang and Rao 2008). Afterwards, Malhotra et al. (2011) conducted a taxonomic revision of *Ovophis* and considered that more than one species of this genus is distributed in Yunnan, while the true *O.monticola* is not found in Yunnan. Subsequently, Zeng et al. (2023) conducted further evaluation of *Ovophis* and described a new species of this genus from Yunnan. However, David et al. (2024) considered that the nomen “*Ovophismalhotrae*” proposed by Zeng et al. (2023) is nomenclaturally unavailable and proposed for it a new nomen. Therefore, four species of *Ovophis* are distributed in Yunnan currently, namely *O.makazayazaya*, *O.zayuensis*, *O.anitae*, and *O.jenkinsi* (Zeng et al. 2023; David et al. 2024; Qiu et al. 2024).

During our recent fieldwork in southern Yunnan, China, some mountain pitviper specimens were collected from Yuanyang Guanyinshan Provincial Nature Reserve. Molecular and morphological comparison revealed that these specimens belong to a distinct taxon in the genus *Ovophis*. Herein, we describe this taxon as a new species.

## ﻿Material and methods

Field surveys were conducted in Yuanyang Guanyinshan Provincial Nature Reserve, Yuanyang County, Honghe Hani and Yi Autonomous Prefecture, Yunnan Province, China, under the permit from Yuanyang Guanyinshan Provincial Nature Reserve Management and Protection Bureau. Specimens were preserved in approximately 75% ethanol and then deposited at Kunming Natural History Museum of Zoology, Kunming Institute of Zoology, Chinese Academy of Sciences (KIZ).

Total genomic DNA was extracted from liver tissues. Fragments of mitochondrial cytochrome *b* (Cytb) gene were amplified and sequenced using the primers L14910 and H16064 (Burbrink et al. 2000). Amplification and sequencing were completed by Sangon Biotech (Shanghai) Co., Ltd. Newly generated sequences have been deposited in GenBank and homologous sequences were obtained from GenBank (Table 1).

**Table 1.** Samples used for the phylogenetic analyses in this study.

Species	Voucher	Locality	GenBank Accession
*Ovophiszhaoermii* sp. nov.	KIZ2023041	Yuanyang, Yunnan, China	PV035804
*Ovophiszhaoermii* sp. nov.	KIZ2024078	Yuanyang, Yunnan, China	PV035805
*Ovophiszhaoermii* sp. nov.	KIZ2024079	Yuanyang, Yunnan, China	PV035806
*Ovophiszhaoermii* sp. nov.	KIZ2024080	Yuanyang, Yunnan, China	PV035807
*Ovophisanitae*	GP 2041	Yunnan, China	OP441841
*Ovophisanitae*	GP 2053	Jinping, Yunnan, China	OP441842
*Ovophisanitae*	GP 2225	Jinping, Yunnan, China	OP441843
*Ovophisanitae*	ROM 39381	Lao Cai, Vietnam	HQ325160
*Ovophisanitae*	ROM 39382	Lao Cai, Vietnam	HQ325161
*Ovophisanitae*	ROM 39384	Lao Cai, Vietnam	HQ325162
*Ovophisanitae*	YBU 19103	Pingbian, Yunnan, China	OP441844
*Ovophisconvictus*	AM B580	Cameron highlands, Pahang, Malaysia	HQ325129
*Ovophisconvictus*	AM B628	Cameron highlands, Pahang, Malaysia	HQ325141
*Ovophisconvictus*	AM B629	Pulau Langkawi, Malaysia	HQ325142
*Ovophisjenkinsi*	IOZ 002680	Yingjiang, Yunnan, China	PP171455
*Ovophisjenkinsi*	IOZ 002679	Yingjiang, Yunnan, China	PP171456
*Ovophisjenkinsi*	CAS 224424	Kachin, Myanmar	HQ325176
*Ovophisjenkinsi*	CAS 234763	Kachin, Myanmar	HQ325179
*Ovophisjenkinsi*	CAS 234866	Kachin, Myanmar	HQ325180
*Ovophisjenkinsi*	GP 1617	Myitkyina, Kachin, Myanmar	OP441885
*Ovophismakazayazaya*	AM A87	Taiwan, China	AF171907
*Ovophismakazayazaya*	AM B480	Yunnan, China	HQ325123
*Ovophismakazayazaya*	AM B482	China	AY352748
*Ovophismakazayazaya*	AM B578	China	HQ325128
*Ovophismakazayazaya*	AM B664	Sichuan, China	HQ325143
*Ovophismakazayazaya*	AM B665	Sichuan, China	HQ325144
*Ovophismakazayazaya*	AM B666	Sichuan, China	HQ325145
*Ovophismakazayazaya*	AM B669	Sichuan, China	HQ325146
*Ovophismakazayazaya*	AM B791	Yunnan, China	HQ325173
*Ovophismakazayazaya*	AM B793	Yunnan, China	HQ325174
*Ovophismakazayazaya*	AM B795	Yunnan, China	HQ325175
*Ovophismakazayazaya*	CAS 23440	Yunnan, China	HQ325178
*Ovophismakazayazaya*	GP 19	Hongya, Sichuan, China	OP441864
*Ovophismakazayazaya*	GP 20	Hongya, Sichuan, China	HQ325165
*Ovophismakazayazaya*	GP 21	Huili, Sichuan, China	OP441856
*Ovophismakazayazaya*	GP 23	Yunnan, China	AY763230
*Ovophismakazayazaya*	GP24/R01	Jingdong, Yunnan, China	HQ325166
*Ovophismakazayazaya*	GP 25	Hongya, Sichuan, China	HQ325167
*Ovophismakazayazaya*	GP 214	Anzhou, Sichuan, China	OP441863
*Ovophismakazayazaya*	GP 227	Hekou, Yunnan, China	HQ325170
*Ovophismakazayazaya*	GP 228	Hekou, Yunnan, China	HQ325171
*Ovophismakazayazaya*	GP 343	Shimen, Hunan, China	HQ325172
*Ovophismakazayazaya*	GP 1030	Yizhang, Hunan, China	OP441848
*Ovophismakazayazaya*	GP 2052	Jiangxi, China	OP441845
*Ovophismakazayazaya*	GP 2544	Hongkou, Sichuan, China	OP441866
*Ovophismakazayazaya*	GP 3831	Qianshan, Jiangxi, China	OP441846
*Ovophismakazayazaya*	GP 5058	Kaihua, Zhejiang, China	OP441847
*Ovophismakazayazaya*	GP 6748	Shangri-La, Yunnan, China	OP441857
*Ovophismakazayazaya*	GP 7749	Xuyong, Sichuan, China	OP441870
*Ovophismakazayazaya*	KIZ 02143	Luquan, Yunnan, China	OP441860
*Ovophismakazayazaya*	KIZ 03100	Youyang, Chongqing, China	OP441853
*Ovophismakazayazaya*	KIZ 037717	Fugong, Yunnan, China	OP441858
*Ovophismakazayazaya*	KIZ 037718	Fugong, Yunnan, China	OP441859
*Ovophismakazayazaya*	KIZ 09136	Pingbian, Yunnan, China	OP441852
*Ovophismakazayazaya*	NTNU B200800	Taiwan, China	DQ305463
*Ovophismakazayazaya*	SCUM035040	Huili, Sichuan, China	AY763229
*Ovophismakazayazaya*	YBU 061033	Anzhou, Sichuan, China	OP441867
*Ovophismakazayazaya*	YBU 091099	Changning, Sichuan, China	OP441871
*Ovophismakazayazaya*	YBU 09115	Changning, Sichuan, China	OP441865
*Ovophismakazayazaya*	YBU 11160	Leishan, Guizhou, China	OP441849
*Ovophismakazayazaya*	YBU 13314A	Shennongjia, Hubei, China	OP441869
*Ovophismakazayazaya*	YBU 13315A	Shennongjia, Hubei, China	OP441874
*Ovophismakazayazaya*	YBU 14212	Lincang, Yunnan, China	OP441854
*Ovophismakazayazaya*	YBU 14281	Mengzi, Yunnan, China	OP441850
*Ovophismakazayazaya*	YBU 15042	Wenshan, Yunnan, China	OP441855
*Ovophismakazayazaya*	YBU 15043	Wenshan, Yunnan, China	OP441851
*Ovophismakazayazaya*	YBU 15186	Muchuan, Sichuan, China	OP441873
*Ovophismakazayazaya*	YBU 15191	Muchuan, Sichuan, China	OP441872
*Ovophismakazayazaya*	YBU 18171	Hekou, Sichuan, China	OP441868
*Ovophismakazayazaya*	YPX 53011	Weixi, Yunnan, China	OP441861
*Ovophismakazayazaya*	YPX 53013	Weixi, Yunnan, China	OP441862
*Ovophismakazayazaya*	ZMB 70214	China	HQ325137
*Ovophismakazayazaya*	ZMB 70219	China	HQ325134
*Ovophismakazayazaya*	ZMB 70220	China	HQ325135
*Ovophismakazayazaya*	ZMB 70221	China	HQ325136
*Ovophismonticola*	ZMB 70216	Gandaki, Nepal	HQ325138
*Ovophismonticola*	ZMB 70218	Gandaki, Nepal	HQ325131
*Ovophismonticola*	GP 2028	Nyalam, Xizang, China	OP441883
*Ovophismonticola*	GP 2050	Nyalam, Xizang, China	OP441884
*Ovophistonkinensis*	AM B581	China	HQ325130
*Ovophistonkinensis*	AM B806	Hainan, China	HQ325181
*Ovophistonkinensis*	GP 1632	Maoming, Guangdong, China	OP441875
*Ovophistonkinensis*	GP 1665	Maoming, Guangdong, China	OP441876
*Ovophistonkinensis*	GP 2140	Lingshui, Hainan, China	OP441877
*Ovophistonkinensis*	GP 7703	Hainan, China	OP441878
*Ovophistonkinensis*	KIZ 011602	Xuan Son, Phu Tho, Vietnam	OP441880
*Ovophistonkinensis*	KIZ 022262	Xing ‘an, Guangxi, China	OP441881
*Ovophistonkinensis*	MVZ 226627	Vinh Phuc, Vietnam	HQ325151
*Ovophistonkinensis*	ROM 29763	Vinh Phuc, Vietnam	HQ325157
*Ovophistonkinensis*	ROM 30776	Gia Lai, Vietnam	HQ325164
*Ovophistonkinensis*	ROM 31082	Vinh Phuc, Vietnam	HQ325158
*Ovophistonkinensis*	ROM 7798	Gia Lai, Vietnam	AY223572
*Ovophistonkinensis*	YPX 28352	Lingshui, Hainan, China	OP441882
*Ovophistonkinensis*	ZMB 70223	China	HQ325132
*Ovophistonkinensis*	ZMB 70224	Lao Cai, Vietnam	HQ325133
*Ovophistonkinensis*	ZMB 70225	China	HQ325139
*Ovophistonkinensis*	ZMB 70226	China	HQ325140
*Ovophiszayuensis*	CAS 233203	Kachin, Myanmar	HQ325177
*Ovophiszayuensis*	GP 89	Mêdog, Xizang, China	OP441887
*Ovophiszayuensis*	GP 90	Zayü, Xizang, China	HQ325168
*Ovophiszayuensis*	GP 92	Gongshan, Yunnan, China	HQ325169
*Ovophiszayuensis*	GP 557	Bayi, Xizang, China	OP441889
*Ovophiszayuensis*	GP 594	Bayi, Xizang, China	OP441893
*Ovophiszayuensis*	GP 611	Bayi, Xizang, China	OP441891
*Ovophiszayuensis*	GP 713	Bomi, Xizang, China	OP441890
*Ovophiszayuensis*	GP 1388	Mêdog, Xizang, China	OP441886
*Ovophiszayuensis*	GP 1505	Zayü, Xizang, China	OP441892
*Ovophiszayuensis*	KIZ 010968	Mêdog, Xizang, China	MW111486
*Ovophiszayuensis*	KIZ 035124	Gongshan, Yunnan, China	OP441895
*Ovophiszayuensis*	KIZ 037721	Lushui, Yunnan, China	OP441894
*Ovophiszayuensi*s	KIZYPX27835	Bomi, Xizang, China	MW133461
*Ovophiszayuensis*	KIZYPX27855	Zayü, Xizang, China	MW133460
*Ovophiszayuensis*	KIZYPX27857	Nyingchi, Xizang, China	MW133459
*Ovophiszayuensis*	YBU 071107	Bayi, Xizang, China	OP441888
Ovophiscf.meridionalis	ROM 39385	Lao Cai, Vietnam	HQ325163
Ovophiscf.meridionalis	ROM 39386	Lao Cai, Vietnam	HQ325154
Ovophiscf.meridionalis	ROM 39387	Lao Cai, Vietnam	HQ325155
Ovophiscf.meridionalis	ROM 39388	Lao Cai, Vietnam	HQ325156
Ovophiscf.tonkinensis	GP 2042	Guangxi, China	OP441879
Ovophiscf.tonkinensis	ROM 35310	Cao Bang, Vietnam	HQ325159
*Ovophis* sp.	FMNH 258632	Xe Kong, Laos	HQ325124
*Ovophis* sp.	FMNH 258633	Champassak, Laos	HQ325125
*Ovophis* sp.	FMNH 258634	Champassak, Laos	HQ325126
*Ovophis* sp.	FMNH 258635	Champassak, Laos	HQ325127
*Ovophis* sp.	ROM 37617	Kon Tum, Vietnam	HQ325152
*Ovophis* sp.	ROM 37618	Kon Tum, Vietnam	HQ325153
*Protobothropsmucrosquamatus*	GP 5683	Cuiping, Sichuan, China	OP441896

Sequences were aligned using MAFFT 7.471 (Katoh and Standley 2013). A Bayesian inference (BI) was performed in MrBayes 3.2.7 (Ronquist et al. 2012) using the GTR+F+I+G4 model and a maximum likelihood (ML) analysis was performed in IQ-TREE 1.6.12 (Nguyen et al. 2015) using the TN+F+I+G4 model, which was selected under the Akaike Information Criterion in ModelFinder (Kalyaanamoorthy et al. 2017). The technical computation methods for BI and ML phylogenetic analyses and genetic divergences calculation were the same as those in Liu et al. (2023).

Measurements were taken with a digital caliper to the nearest 0.1 mm. Measurement methodology followed David and Vogel (2012). Values for symmetric head characters are given in left/right order. The following morphological characteristics were noted:

**Cep** cephalic scales, number on a line between the middle of supraoculars;

**DSR** dorsal scale rows, at one head length behind the head, at midbody (namely at SVL/2), and at one head length before the vent, respectively;

**HL** head length, from the tip of the snout to the angle of the jaw;

**IL** infralabial scales;

**SC** subcaudal plates;

**SL** supralabial scales;

**SVL** snout-vent length;

**TaL** tail length;

**TL** total length;

**VEN** ventral plates.

## ﻿Results

**Figure 1.**
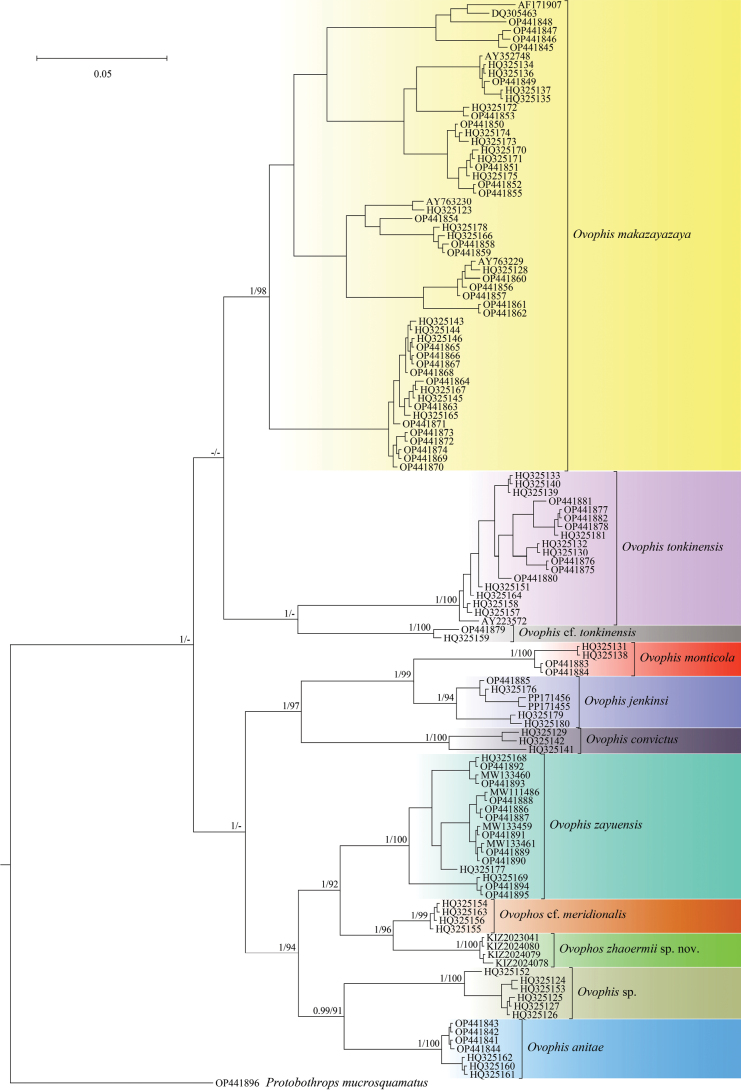
Bayesian phylogram of the genus *Ovophis* inferred from *Cytb* sequences. Numbers after and behind “/” are Bayesian posterior probabilities and ML ultrafast bootstrap values (only values above 0.90/90 are shown), respectively.

The BI and ML analyses yielded a consistent topology, which was almost identical to that published in Zeng et al. (2023). The sequences of the specimens from Yuanyang Guanyinshan Provincial Nature Reserve formed a distinct clade sister to a clade consisting of sequences of specimens from Lao Cai, Vietnam with strong support by both BI and ML, and then they together formed a clade sister to *Ovophiszayuensis* with strong support by BI (Fig. 1). The uncorrected pairwise distance between the sequences of the specimens from Yuanyang Guanyinshan Provincial Nature Reserve and the clade consisting of sequences of specimens from Lao Cai, Vietnam was 4.0%, the uncorrected pairwise distance between the sequences of the specimens from Yuanyang Guanyinshan Provincial Nature Reserve and the sequences of *O.zayuensis* was 7.4%, and the uncorrected pairwise distances between the sequences of the specimens from Yuanyang Guanyinshan Provincial Nature Reserve and the sequences of other species of this genus ranged from 9.1% to 13.0% (Table 2).

**Table 2.** Uncorrected pairwise genetic distances (%) estimated from *Cytb* sequences.

	1	2	3	4	5	6	7	8	9	10
1 *Ovophiszhaoermii* sp. nov.										
2 *Ovophisanitae*	9.1									
3 *Ovophisconvictus*	11.6	10.9								
4 *Ovophisjenkinsi*	11.9	11.5	11.6							
5 *Ovophismakazayazaya*	11.6	10.8	12.7	11.9						
6 *Ovophismonticola*	13.0	12.4	12.2	7.2	13.3					
7 *Ovophistonkinensis*	11.7	10.9	12.4	11.9	11.1	13.7				
8 *Ovophiszayuensis*	7.4	8.5	11.4	10.0	10.8	12.2	11.5			
9 Ovophiscf.meridionalis	4.0	7.5	9.8	10.6	10.3	13.1	10.5	6.2		
10 Ovophiscf.tonkinensis	12.4	10.6	12.9	12.0	10.2	13.8	9.2	11.1	11.0	
11 *Ovophis* sp.	9.6	8.1	12.6	11.4	12.1	12.6	11.8	9.1	8.9	11.3

### ﻿ Ovophis zhaoermii sp. nov.

Taxon classificationAnimaliaSquamataViperidae

41CFF1B7-8657-59CD-9F43-36A577FF7A67

https://zoobank.org/43381C87-E16A-455D-AB2A-DD7F15E88BA2

Figs 2 , 3 , 4

#### Material examined.

***Holotype*.** • KIZ2024078, adult male, collected on 23 July 2024 by Shuo Liu from Yuanyang Guanyinshan Provincial Nature Reserve, Yuanyang County, Honghe Hani and Yi Autonomous Prefecture, Yunnan Province, China (23°1'43"N, 102°56'11"E; 2400 m a.s.l.). ***Paratypes*.** • KIZ2023041, adult male, collected on 16 May 2023, and KIZ2024079–KIZ2024080, two adult males, collected on 16 July 2024, all by Shuo Liu from the same locality as the holotype.

#### Diagnosis.

Ratio of tail length to total length 0.191–0.206, internasals separated by one or two scales, second supralabial bordering loreal pit, dorsal scales in 22-21-17 rows, ventrals 146–148, subcaudals 57–64, 3–11 subcaudals unpaired and other subcaudals paired, third supralabial larger than fourth, dorsal surface of head unpatterned, dorsal surface of body brownish-black or reddish-brown with rectangular black blotches, series of white spots on dorsal surface of tail continuous, iris off-white with a black mesh pattern.

**Figure 2.**
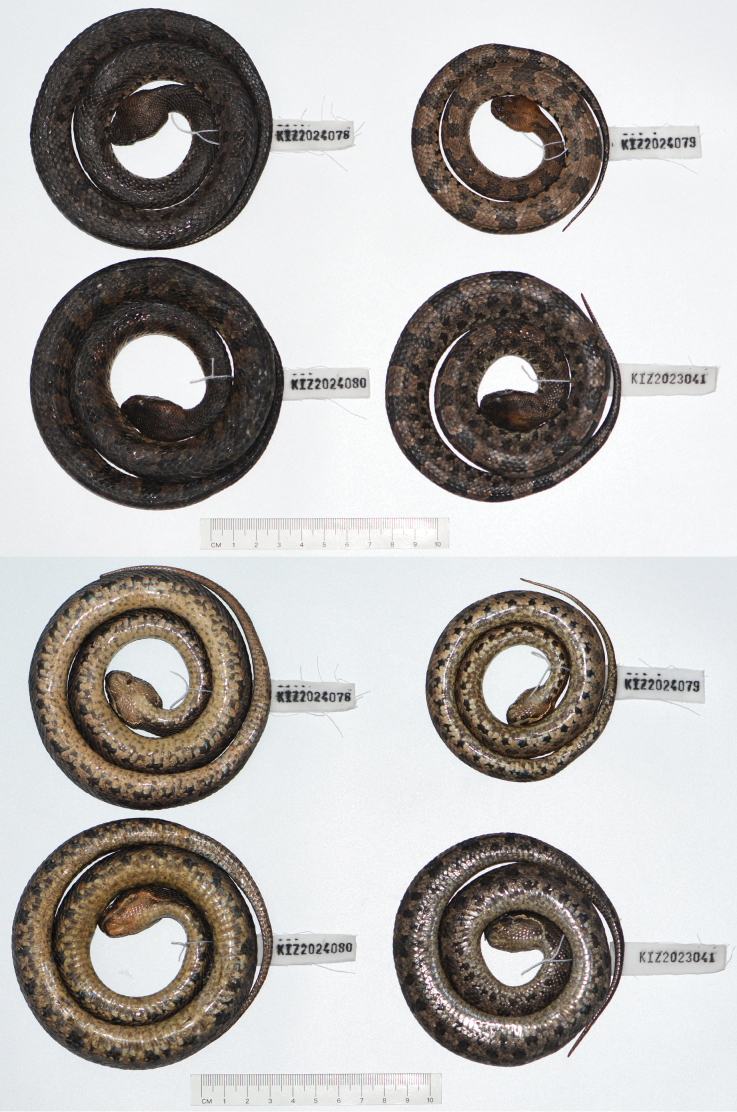
Dorsal view (top) and ventral view (bottom) of the type series of *Ovophiszhaoermii* sp. nov. in preservative.

#### Description of holotype.

Adult male; body relatively slender, tail relatively short, SVL 541 mm, TaL 133 mm, TaL/TL 0.197; head approximately pear shaped, distinct from neck, HL/SVL 0.05; snout blunt and rounded, rostral trapezoidal, upper tip slightly visible from above; eye small, pupil vertically elliptic; supraocular 1/1, elongate, oval, largest scales on dorsal head, separated by six small scales; nostril close to snout tip; two internasals, approximately rectangular, separated by one small scale; two much smaller scales between rostral and internasals; loreal 1/1; preoculars 3/3; postoculars 2/3; suboculars 1/3, separated from supralabials by two rows of scales; supralabials 8/9, first and second in contact with nasal, second bordering loreal pit, third larger than fourth; infralabials 9/10, first pair contacting each other behind mental, first to third in contact with chin shields; mental triangular; one pair of chin shields, meeting in midline; dorsal scales in 22-21-17 rows, distinctly keeled except outer row; ventral scales 148, excluding four preventrals; subcaudal scales 61, first to eleventh unpaired, others paired; cloacal plate undivided.

**Figure 3.**
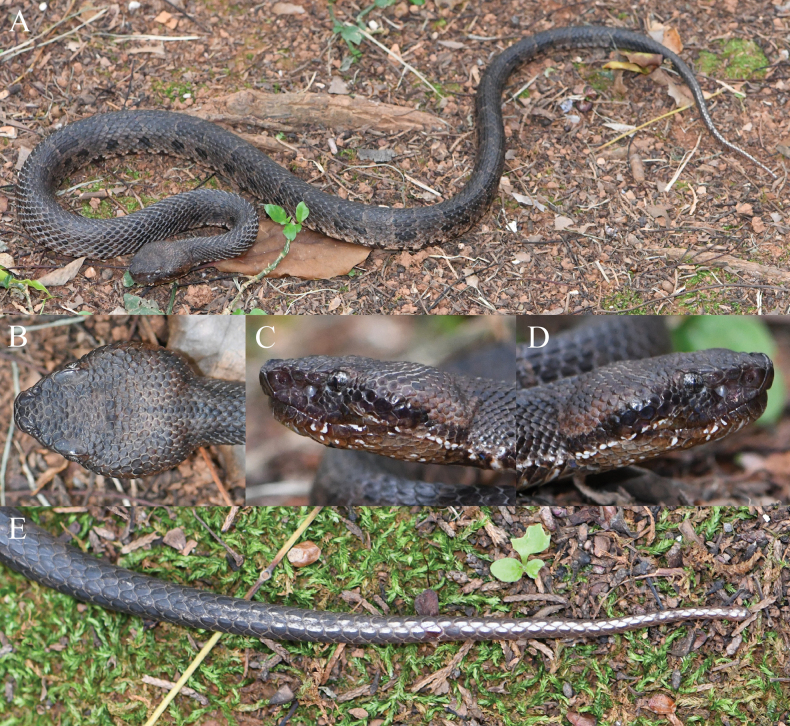
The holotype (KIZ2024078) of *Ovophiszhaoermii* sp. nov. in life **A** general view **B** dorsal view of the head **C** left view of the head **D** right view of the head **E** dorsal view of the tail.

***Color of holotype in life*.** Dorsal surface of head brownish-black; lateral surface of head dark reddish-brown, a wide brownish-black stripe behind eye on each side, a narrow discontinuous white stripe from mouth corner to lateral neck on each side; lower lip reddish-brown with some irregular white blotches; dorsal surface of body brownish-black with many rectangular, large black blotches on dorsolateral surface, blotches of left and right sides arranged approximately in staggered pattern; two black spots below each black dorsolateral blotch; some irregular black blotches on ventrolateral part of body; anterior dorsal surface of tail dark brownish-black, posterior dorsal surface of tail white, composed of a continuous series of white spots on two medial rows of scales; ventral surface of head reddish-brown with some irregular white blotches; ventral surface of body and tail brownish-yellow with many irregular grayish-brown blotches; iris off-white with a black mesh pattern.

**Figure 4.**
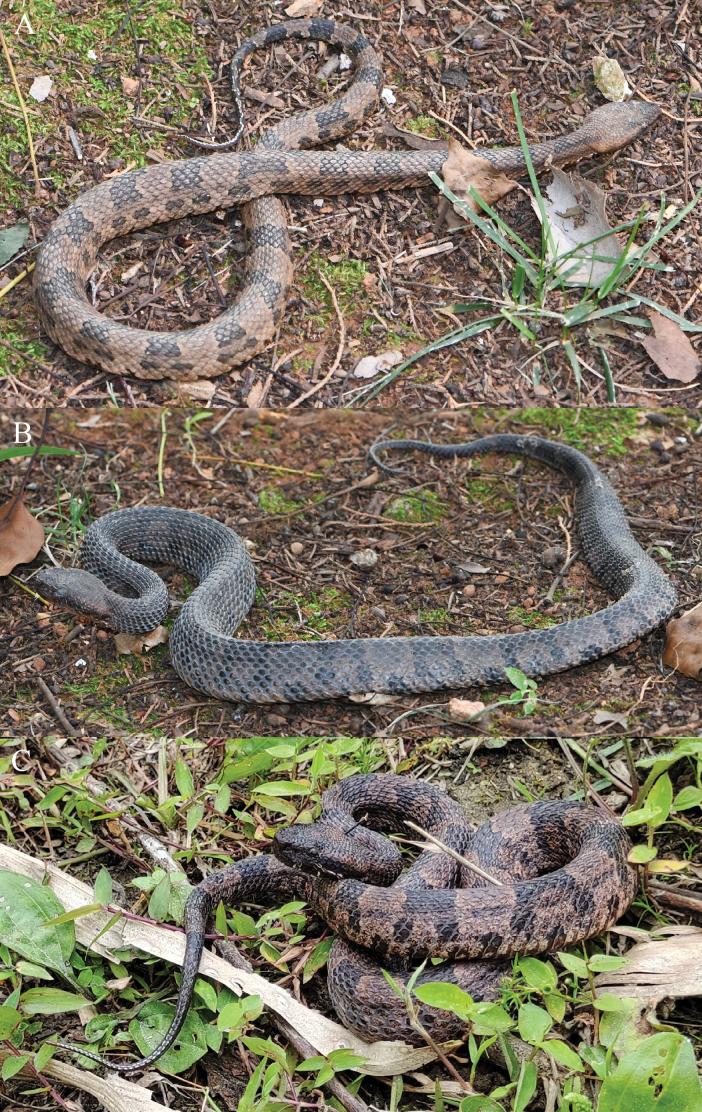
The paratypes of *Ovophiszhaoermii* sp. nov. in life **A** KIZ2024079 **B** KIZ2024080 **C** KIZ2023041.

#### Variations.

Morphometric and meristic data of the type series are provided in Table 3. The paratypes resemble the holotype in most aspects except for small differences in body size, relative tail length, and the number of unpaired subcaudals. In addition, the larger paratype (KIZ2024080) resembles the holotype in coloration, while the two smaller paratypes (KIZ2023041 and KIZ2024079) have lighter body coloration than the holotype.

**Table 3.** Measurements (in mm) and scalation data of the type specimens of *Ovophiszhaoermii* sp. nov.

	KIZ2024078	KIZ2024079	KIZ2024080	KIZ2023041
	Holotype	Paratype	Paratype	Paratype
	Male	Male	Male	Male
SVL	541	396	543	461
HL	26.7	21.0	27.6	25.0
TaL	133	103	132	109
TL	674	499	675	570
TaL/TL	0.197	0.206	0.196	0.191
Cep	6	6	7	7
DSR	22-21-17	22-21-17	22-21-17	22-21-17
SL	8/9	9/9	8/8	8/8
IL	9/10	10/10	10/10	9/10
VEN	148	147	147	146
SC	61 (1^st^–11^th^ unpaired)	64 (4^th^–11^th^ unpaired)	61 (4^th^–6^th^ unpaired)	57 (15^th^–23^th^ unpaired)

#### Ecology notes.

The specimens of the new species were found on the ground beside a stream at night. No other reptile species were found at the type locality of the new species, but many amphibian species were found in sympatry, including *Amolopsminutus* Orlov & Ho, 2007, *A.viridimaculatus* (Jiang, 1983), *Atympanophrysgigantica* (Liu, Hu & Yang, 1960), *Feihylafuhua* Fei, Ye & Jiang, 2010, *Hylaannectans* (Jerdon, 1870), *Leptobrachiumailaonicum* (Yang, Chen & Ma, 1983), *Nanoranaaenea* (Smith, 1922), and *Zhangixalusduboisi* (Ohler, Marquis, Swan & Grosjean, 2000). Therefore, we presume the new species may prey on frogs in the wild.

#### Distribution.

**Figure 5.**
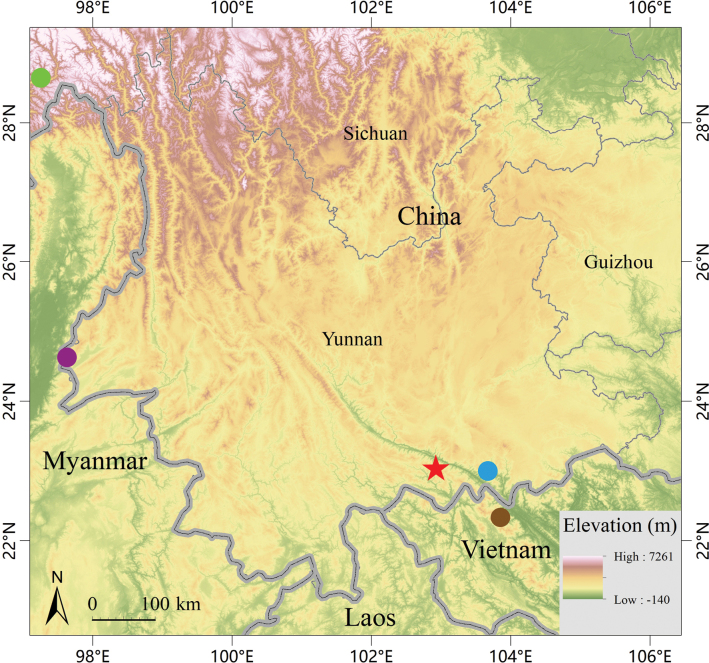
Map showing the type localities of *Ovophiszhaoermii* sp. nov. (red star), *O.anitae* (blue dot), *O.jenkinsi* (purple dot), *O.zayuensis* (green dot), and *Trimeresurusmonticolameridionalis* (brown dot).

The new species is currently known only from Yuanyang Guanyinshan Provincial Nature Reserve in Yuanyang County, Honghe Hani and Yi Autonomous Prefecture, Yunnan Province, China (Fig. 5).

#### Etymology.

Named after the renowned Chinese herpetologist, Prof. Ermi Zhao (1930–2016). The designation of this specific epithet honors his great contribution to herpetological research in China, especially in snake research. According to the type locality of this species, we suggest the English common name “Guanyinshan mountain pitviper” and the Chinese common name “观音山烙铁头蛇 (Pinyin: guān yīn shān lào tiě tóu shé)”.

#### Comparisons.

*Ovophiszhaoermii* sp. nov. can be differentiated from *O.anitae* by having a relatively longer tail (TaL/TL 0.191–0.206 vs 0.133), having more subcaudal scales (57–64 vs 47), some of the subcaudal scales being unpaired (vs all of the subcaudal scales being paired), dorsal scales being in 22-21-17 rows (vs 27-23-19 rows), and the second supralabial bordering the loreal scale (vs the second supralabial being separated from the loreal scale).

*Ovophiszhaoermii* sp. nov. can be differentiated from *O.convictus* by having a relatively longer tail (TaL/TL 0.191–0.206 vs 0.064–0.128), having more subcaudal scales (57–64 vs 17–31), having more ventral scales (146–148 vs 120–140), some of the subcaudal scales being unpaired (vs all of the subcaudal scales being paired), and having continuous white spots on the dorsal surface of the tail (vs scattered white spots on the dorsal surface of the tail).

*Ovophiszhaoermii* sp. nov. can be differentiated from *O.makazayazaya* by having more subcaudal scales (57–64 vs 34–52), some of the subcaudal scales being unpaired (vs all of the subcaudal scales being paired), the third supralabial being larger than the fourth (vs the fourth supralabial being larger than the third), and having continuous white spots on the dorsal surface of the tail (vs scattered white spots on the dorsal surface of the tail).

*Ovophiszhaoermii* sp. nov. can be differentiated from *O.monticola* by having an unpatterned dorsal head surface (vs patterned dorsal head surface), having relatively more subcaudal scales (57–64 vs 37–58), some of the subcaudal scales being unpaired (vs all of the subcaudal scales being paired), the dorsal scales being in 22-21-17 rows (vs 23 or 21-23 or 21-19 rows), and having continuous white spots on the dorsal surface of the tail (vs scattered white spots on the dorsal surface of the tail).

*Ovophiszhaoermii* sp. nov. can be differentiated from *O.jenkinsi* by having a relatively longer tail (TaL/TL 0.191–0.206 vs 0.132–0.184), having more subcaudal scales (57–64 vs 40–52), having more ventral scales (146–148 vs 134–142), some of the subcaudal scales being unpaired (vs all of the subcaudal scales being paired), having an unpatterned dorsal head surface (vs patterned dorsal head surface), and having continuous white spots on the dorsal surface of the tail (vs scattered white spots on the dorsal surface of the tail).

*Ovophiszhaoermii* sp. nov. can be differentiated from *O.tonkinensis* by having more subcaudal scales (57–64 vs 39–49), having more ventral scales (146–148 vs 128–134), most of the subcaudal scales being paired (vs all or most of the subcaudal scales being unpaired), and the third supralabial being larger than the fourth (vs the fourth supralabial being larger than the third).

*Ovophiszhaoermii* sp. nov. can be differentiated from *O.zayuensis* by having fewer ventral scales (146–148 vs 160–177), most of the subcaudal scales being paired (vs all or most of the subcaudal scales being unpaired), the dorsal scales being in 22-21-17 rows (vs 25 or 27-23-19 or 17 rows), and having continuous white spots on the dorsal surface of the tail (vs no visible white spots on the dorsal surface of the tail).

## ﻿Discussion

In the phylogenetic analysis, the sequences which were regarded as *Ovophistonkinensis* by Zeng et al. (2023) formed two highly divergent clades, with a genetic distance of up to 9.2%. The first clade contained some sequences of specimens from southeastern China and northern and central Vietnam, which cover the type locality of *O.tonkinensis*, while the second clade contained two sequences of specimens from Guangxi of China and Cao Bang of Vietnam, respectively. Therefore, we consider that the first clade is *O.tonkinensis*, while the second clade may represent a cryptic species, and we temporarily refer to the second clade as Ovophiscf.tonkinensis.

Qiu et al. (2024) described *Ovophisjenkinsi* from Tongbiguan Township, Yingjiang County, Yunnan Province, China, which is closely related to *O.monticola*, based on morphological and molecular data. However, Qiu et al. (2024) did not include all available sequences of *O.monticola* in their phylogenetic analysis. In this study, we integrated all available sequences of *O.monticola* and the sequences of *O.jenkinsi*, and the analysis showed that among the sequences previously considered to belong to *O.monticola*, those corresponding to specimens from Gandaki of Nepal and Nyalam of China formed one clade, while those corresponding to specimens from Kachin of Myanmar formed another clade together with the sequences of *O.jenkinsi*; the genetic distance between these two clades was 7.2%. Therefore, according to the type locality of *O.monticola*, we consider the clade containing sequences of specimens from Nepal and Nyalam of China to be *O.monticola*, and the other clade to be *O.jenkinsi*. That is to say, *O.monticola* is restricted to Nepal and adjacent southern Xizang of China and northern India, while *O.jenkinsi* is also distributed in northern Myanmar in addition to western Yunnan.

David et al. (2024) considered that *Trimeresurusmonticolameridionalis* Bourret, 1935 may be a distinct species, and it is likely that either *Ovophisanitae* or the *Ovophis* sp. 1 in Zeng et al. (2023) is a junior synonym of *T.m.meridionalis*. It can be confirmed that *Ovophiszhaoermii* sp. nov. is definitely not a junior synonym of *T.m.meridionalis*, although in both species the third supralabial is larger than the fourth one. *Ovophiszhaoermii* sp. nov. differs from *T.m.meridionalis* by having relatively more subcaudal scales (57–64 vs 47–54), having more ventral scales (146–148 vs 134–136), some of the subcaudal scales being unpaired (vs all of the subcaudal scales being paired), the dorsal scales being in 22-21-17 rows (vs 25-25-19 or 23-23-17 rows), and having continuous white spots on the dorsal surface of the tail (vs no visible white spots on the dorsal surface of the tail). However, it cannot be determined whether *O.anitae* or the *Ovophis* sp. 1 in Zeng et al. (2023) is conspecific with *T.m.meridionalis* currently. Until there is sufficient evidence, we retain the validity of *O.anitae* and refer to the *Ovophis* sp. 1 in Zeng et al. (2023) as Ovophiscf.meridionalis for the time being.

The area where *Ovophiszhaoermii* sp. nov. was discovered is located within Yuanyang Guanyinshan Provincial Nature Reserve, which is far away from human settlements. There are intact primary forests in the nature reserve, and they are legally protected. Therefore, we consider that this species is currently not threatened by humans.

## Supplementary Material

XML Treatment for Ovophis zhaoermii

## ﻿Additional information

### Author ORCIDs

Shuo Liu https://orcid.org/0000-0001-7825-3006

Mian Hou https://orcid.org/0000-0002-3322-6570

Dingqi Rao https://orcid.org/0000-0003-2680-1503

Song Li https://orcid.org/0000-0003-0834-5882

### Data availability

All of the data that support the findings of this study are available in the main text.
